# Quality of life in Chinese family caregivers for elderly people with chronic diseases

**DOI:** 10.1186/s12955-016-0504-9

**Published:** 2016-07-06

**Authors:** Hui Xie, Cheng Cheng, Yisheng Tao, Jie Zhang, Delprino Robert, Jihui Jia, Yonggang Su

**Affiliations:** School of Nursing, Shandong University, Jinan, 250012 Shandong China; Nursing Department, Bengbu Medical College, Bengbu, 233030 Anhui China; School of Foreign Languages and Literature, Shandong University, 44 West Wenhua Road, Jinan, 250012 Shandong Peoples Republic of China; School of Public Health, Shandong University, Jinan, 250012 Shandong China; Department of Sociology, State University of New York Buffalo State, Buffalo, NY USA; Department of Psychology, State University of New York Buffalo State, Buffalo, NY USA

**Keywords:** Family caregiver, Quality of life, Chronic diseases, Elderly, Activities of daily living

## Abstract

**Background:**

Inadequate studies have been conducted in China to examine quality of life in family caregivers. Quality of life in family caregivers for elderly people with chronic diseases was evaluated, and the demographic and characteristic factors of both elderly people and their caregivers were explored.

**Methods:**

The 36-Item Short Form Health Survey (SF-36) was used to assess health-related quality of life in 407 family caregivers caring for elderly people with chronic diseases in six communities on the Mainland China. The explanatory variables included family caregivers’ demographic and other caregiving variables related to eldercare. Descriptive statistics and multiple linear regression analysis were used in the data analysis, performed via SPSS 17.0.

**Results:**

Mean SF-36 and physical and mental component scores were 66.14 ± 17.50, 70.06 ± 16.49, and 62.22 ± 18.51, respectively. The scores of caregivers’ physical function and bodily pain were significantly higher, while the scores of caregivers’ role limitations due to physical problems, general health, vitality, social function, mental health and role limitations due to emotional problems were significantly lower. Caregivers’ ages, comorbidity, the perceived effects of caregiving on caregivers’ social lives and elderly individuals’ ages, marital status and Activities of Daily Living scores were significantly associated with the physical component score. In addition, caregivers’ age, the affordability of the elderly person’s healthcare expenses, the perceived effects of caregiving on caregivers’ social lives, and elderly people’s marital status and ADL scores were significantly associated with the mental component score.

**Conclusion:**

Family caregivers for elderly people with chronic diseases showed poorer mental and better physical well-being. Factors of both elderly people and their caregivers impact the caregivers’ quality of life. These findings highlight the importance of addressing mental health of family caregivers, and of providing economical support and psychological care for them.

**Electronic supplementary material:**

The online version of this article (doi:10.1186/s12955-016-0504-9) contains supplementary material, which is available to authorized users.

## Background

Confronted with nation-wide aging problems and influenced by traditional culture, more and more Chinese people are undertaking the task of caring their aging parents or other elderly members. Currently, on the mainland China, approximately one fifth of the total population is aged 60 years and older, accounting more than 200 million in total. This segment of the population will increase to 400 million within 10 years [1]. Nearly 60 % of elderly Chinese people are estimated to have experienced various chronic diseases [2]. The risk of disability in the elderly population increases with the development of chronic diseases, which often increases the demand for special care and support [3]. Due to profound influence of Confucian philosophy whose cultural norm holds that the elderly should be honored and respected and thus be cared in their own homes and by their family members, a considerable proportion of elderly Chinese people choose to receive support and care from family caregivers [1].

Family caregivers are family members who provide a minimum of 1 h of daily care for at least 3 months for care-recipients [4]. A spouse, child, relative, neighbor, or friend could be a caregiver. The potential gains for a caregiver can include positive factors such as self-satisfaction, reciprocity, and the sense of duty having been fulfilled [2,3,5–7]. The activities involved in caring for a chronically ill person could also exert a negative impact on caregiver’s health [8]. However, family caregivers’ health status influences the quality of care for elderly people, similarly, providing home care for someone with a chronic disease can impact caregivers’ physical and psychological health [9]. A cross-sectional study involving hemodialysis patients and their caregivers reported that one third of caregivers were moderately to severely depressed [10]. Gaskamp found that reduction in home health care was associated with decreased quality of life and increased depression among family caregivers of patients [11]. Another study found that caregivers were significantly more likely to display depressive symptoms and meet the diagnostic cutoff for depression relevant to non-caregivers (40 % for caregivers versus 5 % for non-caregivers). Also, one quarter of the targeted caregivers reported taking antidepressant medication for depression [12]. Heesoo et al. found that the number of incremental and informal caregiving hours attributable to stroke among the elderly was 8.5 per patient per week [13]. In China, a study conducted in Beijing reported that the prevalence rate for depressive symptoms in elderly patients’ family caregivers was 51.2 % [14]. Peng conducted a qualitative study of family caregivers for disabled elderly people in Guangzhou, and showed that daily tasks as well as psychological and economic pressures affected caregivers’ physical and mental health, work performance and quality of life [15].

Relative to evaluating the health status of family caregivers from a certain dimension, quality of life can be used to measure the comprehensive status of family caregivers. Quality of life (QoL) is a measurement of the quality of daily life including emotional, social, and physical factors [16]. QoL ratings have been found to be an appropriate metric for the design and implementation of service plans [17]. The International Working Group for the Harmonization of Dementia Guidelines has recommended that QoL be included as an outcome measure in all dementia trials [18].

Previous investigations focused mainly on burden [19,20], psychological health [21], and QoL in family caregivers for elderly patients with dementia [22]. Literature review showed that few studies have been conducted to examine QoL of family caregivers in China and to the research on inpatients. In addition, most did not examine the impact of elderly care recipients to caregivers. Therefore, this study aimed to (1) examine QoL in family caregivers for elderly people with chronic diseases, using the 36-Item Short Form Health Survey (SF-36), and (2) explore the demographic and characteristic factors of both elderly people and their caregivers.

## Methods

### Sampling

A cross-sectional study was carried out with a convenience sample of 6 community health centers from 3 districts from Bengbu, an industrial city with a moderate economic level in Anhui Province in central China. Based on the official records, there are 335,869 residents aged 60 years and above in the city and the mean family income is about 2,197 RMB a month [23]. At the recruitment stage, an invitation letter which explained the purpose of the study was sent to each family, inviting the family caregiver to participate in the study. Family caregivers were eligible to participate if they were 18 years or older, provided home care for a family member older than 60 years of age (according to the standard definition of older persons in developing countries [24]) with one or more chronic diseases, able to communicate in Chinese, and provided home care for more than 5 h per week for more than 3 months [25].

**Fig. 1:**
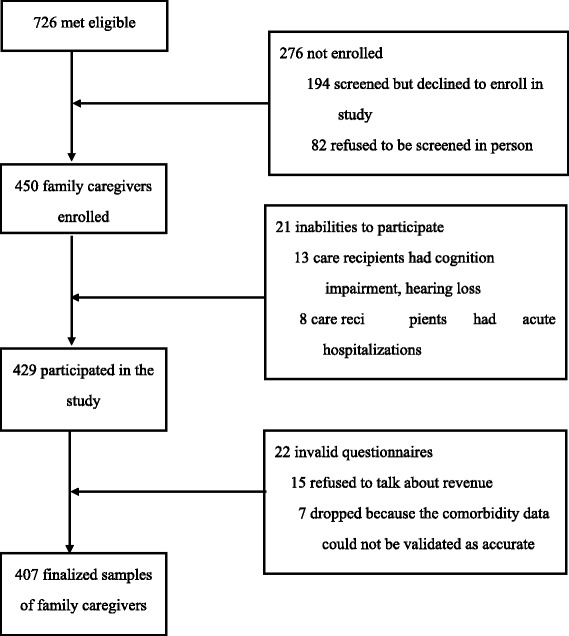
Numbers of family caregivers who were screened, enrolled, and completed study

Of the 726 eligible referrals, 38 % refused to participate, primarily reporting lack of time or interest as major reasons. Thus, a total of 450 family caregivers were enrolled in this study. For the care recipients included in the study, 4.7 % had cognition impairment, hearing loss or acute hospitalizations. The sample at the completion of data collection decreased from 450 to 429. Of those 5.1 % were dropped from the sample due to invalid questionnaires they provided at one of the data collection points. The final sample used for analysis consisted of 407 family caregivers (see Fig. 1).

### Measurement Instruments

#### Quality of life

The primary outcome variable, Quality of Life (QoL), was measured using the 36-Item Short Form Health Survey (SF-36), which was designed to allow self-evaluation of quality of life and summarized the concept of health. It is an extensively applicable scale, and the Chinese version was developed by Ware [26]. The SF-36 consists of 36 questions and two summary scores: physical and mental component scores (PCS and MCS respectively). The PCS consists of the following dimensions: physical function (PF), role limitations due to physical problems (RP), bodily pain (BP), and general health (GH). The MCS consists of the following dimensions: vitality (VT), social function (SF), mental health (MH), and role limitations due to emotional problems (RE) [27]. Individual item scores are summed up and transformed into a 0–100 scale ranging from the worst possible QoL to the best possible QoL [28]. Cronbach’s α for the current sample was 0.88.

#### Demographic and health characteristics of caregivers and care recipients

Caregivers’ demographic and other characteristics included gender, age, marital status, educational level, occupation, monthly revenue, and comorbidity. Explanatory variables for elderly people with chronic diseases included functional status and demographic characteristics including gender, age, marital status, educational level, healthcare insurance, and comorbidity. Care recipients’ functional status was measured via the Activities of Daily Living (ADL) Scale, which includes Physical [29] and Instrumental Activities of Daily Living (ADL and IADL respectively) subscales [30]. The ADL subscale assesses 6 types of ability: bathing, dressing, using the toilet, transfer, eating, and others. The IADL subscale assesses the ability to perform 8 types of more complex activities, such as using the phone or transportation and going shopping. Scores for ability to perform activities range from 1 to 4 (1 point for each activity performed without help and 4 points for each activity that the individual is unable to perform). The maximum score is 56 (higher scores indicate greater dependence). Ramos validated the ADL scales in a Brazilian sample, demonstrating Cronbach’s alpha of 0.88 [30]. Elderly people were classified into 3 categories according to degree of dependence: independent (<16 points), partially dependent (16–22 points), and severely dependent (a total score of >22 or more than 2 items with scores of ≥3) [31]. Cronbach’s α for the current sample was 0.93.

#### Caregiving factors

Caregiving factors examined in the study included the relationship to the elderly person (spouse, child, parent, or other), overall caregiving duration, average daily caregiving duration, affordability of the elderly person’s healthcare expenses (≥90, 89–50 %, 49–11 %, ≤10 %), and importance of the perceived effects of caregiving on the caregiver’s social life (important, relatively important, slightly important, or not important at all).

### Procedure

Approval was obtained from the 6 community health centers. Data were elicited primarily via in-person interviews with caregivers and care recipients conducted by bachelor’s prepared research assistants with specialized preparation in the use of the questionnaires and data collection processes provided by the research team. The research assistants visited the participants’ homes and explained the purpose of the study, seeking voluntary participation. Consent was obtained from the caregivers and care recipients who agreed to participate. The comorbidity data of caregivers and care recipients were obtained by research assistants from enrollees’ medical records.

### Statistical analysis

All questionnaire responses were recorded using Epidata 3.1 [32], and analyses were performed using SPSS version 17 (SPSS Inc., Chicago, IL, USA) statistical software. Caregivers’ and elderly individuals’ characteristics were expressed as frequencies or percentages for categorical variables and means with standard deviations for continuous variables. Overall SF-36 scores, the PCS and MCS, and scores for the 8 subscales of the SF-36 were calculated using scoring algorithms. A single-sample t test was performed to estimate the differences between caregivers’ scores and Chinese general population norms. Multivariate linear regression analyses were performed, using the PCS and MCS as separate dependent variables. Variables concerning several demographic and other characteristics for caregivers and the elderly were entered as independent variables.

## Results

### Participant characteristics

Table 1 shows the three main types of data collected, the first of which includes characteristics of both family caregivers and the elderly. Elderly care recipients’ minimum and maximum ADL scores were 17 and 54, respectively. Their average ADL score was 21.05, with 294 and 113 elderly people classed as partially and severely dependent, respectively. The prevalence rate for hypertension in elderly people with chronic disease was 29.6 %, and coronary disease 9.9 %, diabetes mellitus 9.6 %, rheumatic arthritis 9.5 %, cerebrovascular disease 9.2 % etc. (see Additional file 1) In addition, 379 (93.1 %) elderly people had medical insurance, which included 123 (30.2 %) urban resident basic medical insurance and 167 (41.0 %) rural cooperative medical insurance.

**Table 1:** Characteristics of Caregivers and Elderly People with Chronic Diseases, and Descriptive Statistics (*n* = 407)

Characteristic	Caregiver	Elderly
	*n* (%)	*n* (%)
Gender		
male	175 (43.0)	168 (41.3)
female	232 (57.0)	239 (58.7)
Age		
20–39	65 (16.0)	
40–59	229 (56.2)	
60–69	113 (27.8)	153 (37.6)
70–79		192 (47.2)
80+		62 (15.2)
Marital status		
married	381 (93.6)	284 (69.8)
unmarried/widowed/separation	26 (6.4)	123 (30.2)
Education level		
illiterate	45 (11.0)	160 (39.3)
elementary school	67 (16.5)	125 (30.7)
secondary school	146 (35.9)	82 (20.2)
high school or professional training	98 (24.1)	31 (7.6)
college and above	51 (12.5)	9 (2.2)
Occupation		
employed	207 (50.8)	
retired	89 (21.9)	205 (50.4)
never been employed or others	111 (27.3)	202 (49.6)
Average monthly revenue (RMB)		
≤500	26 (6.4)	
501–1000	78 (19.2)	
1001–2000	114 (28.0)	
2001–3000	104 (25.5)	
>3000	85 (20.9)	
Comorbidity (hypertension, coronary disease, diabetes mellitus, etc.)		
none	265 (65.1)	
one	103 (25.3)	107 (26.3)
two	23 (5.7)	103 (25.3)
three and more	16 (3.9)	197 (48.4)
Activities of Daily Living		
independent		0 (0.0)
partially dependent		294 (72.2)
severely dependent		113 (27.8)
Relationship to the elderly person		
spouse	109 (26.8)	
children	201 (49.4)	
children in-law	80 (19.7)	
other relatives	17 (4.1)	
Overall caregiving duration (year)		
<1	112 (27.5)	
1–2	88 (21.6)	
3–5	87 (21.4)	
6–9	48 (11.8)	
10+	72 (17.7)	
Average daily caregiving duration (hours)		
<2	178 (43.8)	
2–3	127 (31.2)	
4–5	71 (17.4)	
6+	31 (7.6)	
Affordability of the elderly person’s healthcare expenses		
≥90 %	43 (10.5)	
89 %–50 %	183 (45.0)	
49 %–11 %	151 (37.1)	
≤10 %	30 (7.4)	
Importance of the perceived effects of caregiving on the caregiver’s social life		
important	55 (13.5)	
relatively important	186 (45.7)	
slightly important	147 (36.1)	
not important at all	19 (4.7)	

### QoL in Participants and the General Population

The results for the eight dimensions of the SF-36 are presented in Table 2. Mean values and standard deviation for the overall SF-36 score, PCS, and MCS were 70.06 ± 16.49; 62.22 ± 18.51, and 66.14 ± 17.50, respectively. Caregivers’ PF and BP were significantly higher relative to the Chinese national norms, which were calculated using 17,754 study subjects who were randomly selected from six cities of China [33]. In addition, caregivers’ RP, GH, VT, SF, RE, and MH scores were significantly lower relative to those of the general population. The reduction in caregivers scores was approximately 25 points below the scores for RP and RE norms and approximately 10 points below that for GH.

**Table 2:** Comparison of scores for the eight dimensions of the SF-36 between caregivers and the general population

	Caregiver	General population	*t*	*P*
	Mean ± SD	Mean ± SD		
Physical Function (PF)	90.65 ± 8.53	87.92 ± 16.98	4.397	<0.001
Role Physical (RP)	52.64 ± 34.39	77.50 ± 34.86	−14.581	<0.001
Bodily Pain (BP)	84.60 ± 9.91	82.22 ± 16.98	2.408	0.016
General Health (GH)	52.36 ± 13.15	62.51 ± 17.88	−15.570	<0.001
Vitality (VT)	66.52 ± 10.39	68.17 ± 17.63	−3.196	0.002
Social Function (SF)	75.71 ± 16.71	80.67 ± 19.98	−5.935	<0.001
Role Emotional (RE)	40.96 ± 35.09	67.86 ± 39.44	−15.220	<0.001
Mental Health (MH)	65.67 ± 11.86	68.47 ± 16.90	−4.762	<0.001

### Factors Significantly Associated with Family Caregivers’ QoL

After the explanatory variables associated with family caregivers’ SF-36 scores had been considered, caregivers’ age and comorbidity, recipients’ age, marital status, and ADL scores, and the perceived effects of caregiving on caregivers’ social lives were significantly associated with the PCS (Table 3). Of these 6 variables, caregivers’ age explained the largest proportion of the variance in the PCS (PCS model: *F* = 9.428, *P* = 0.000, *R* = 0.577, *R*^2^ = 0.333, adjusted *R*^2^ = 0.298, SE = 12.167). For the MCS scale, caregivers’ age and comorbidity, care recipients’ marital status and ADL scores, the affordability of the elderly person’s healthcare expenses, and the perceived effects of caregiving on caregivers’ social lives were significantly associated with the MCS. Of these variables, the perceived effects of caregiving on caregivers’ social lives explained the largest proportion of the variance in the MCS (MCS model: *F* = 7.498, *P* = 0.000, *R* = 0.533, *R*^*2*^ = 0.285, adjusted *R*^2^ = 0.247, and SE = 11.080).

**Table 3:** Multivariate models of factors concerning caregivers and the elderly for the PCS and MCS in caregivers for the elderly (*n* = 407)

variables	PCS MCS			
	β (SE)	β^a^	β (SE)	β^a^
Caregivers’ demographic factors				
age	−0.389 (0.073)	−0.337^*^	−0.201 (0.069)	−0.198^*^
gender	−0.308 (1.243)	−0.011	−1.099 (1.175)	−0.043
marital status	2.766 (2.091)	0.056	2.328 (1.976)	0.053
education level	−0.493 (0.618)	−0.042	−0.392 (0.584)	−0.038
occupation	−0.030 (0.417)	−0.003	0.067 (0.394)	0.008
average monthly revenue	0.746 (0.569)	0.061	0.220 (0.538)	0.020
comorbidity	−5.630 (0.874)	−0.301^*^	−2.833 (0.826)	−0.172^*^
Elderly’ demographic factors				
age	−0.217 (0.108)	−0.102^*^	−0.083 (0.102)	−0.044
gender	1.538 (1.321)	0.053	0.654 (1.248)	0.025
marital status	−1.639 (0.725)	−0.103^*^	−1.774 (0.685)	−0.126^*^
education level	−0.291 (0.624)	−0.022	−0.575 (0.589)	−0.049
ADL	−0.203 (0.096)	−0.107^*^	−0.327 (0.091)	−0.195^*^
Caregiving factors				
relationship to the elderly person	−0.173 (0.706)	−0.015	−0.930 (0.667)	−0.091
overall caregiving duration	0.391 (0.454)	0.038	0.027 (0.429)	0.003
average daily caregiving duration	1.049 (0.772)	0.068	0.958 (0.729)	0.071
affordability of the elderly person’s healthcare expenses	1.567 (0.861)	0.083	2.490 (0.813)	0.150^*^
importance of the perceived effects of caregiving on the caregiver’s social life	5.185 (0.915)	0.274^*^	4.681 (0.865)	0.282^*^

β represents estimated regression coefficient, standing for the mean difference between the index and reference categories and standing for the average increase (decrease) in 2 scales of the SF-36 for each 1-unit increase for continuous variables; SE represents standard error

^a^ Standardized regression coefficient; ^*^ *P* < 0.05

## Discussion

### Participants’ QoL

The present study examined QoL of caregivers for elderly people with chronic diseases, with subjective assessment of well-being and factors concerning both caregivers and the elderly. The findings of this study indicated that there was a substantial difference between caregivers and the general population; family caregivers displayed superior PF and fewer reports of BP relative to the general population. These findings are likely to have occurred because most caregivers (56.2 %) were aged 40–60 years, 50.8 % were employed, and 65.1 % did not have chronic diseases. In addition, superior physical functioning may have been one of the reasons why these caregivers cared for their elderly relatives [34]. Further, caregivers displayed lower values for the MCS. The burden and stress of fulfilling both family and work commitments were sufficiently severe to affect the psychological and social aspects of their personal quality of life, particularly with respect to RE, RP, and GH.

Another Asian study found that primary caregivers for elderly people with chronic diseases showed poorer mental and better physical well-being relative to population norms in Taiwan [35]. Relative to their results, scores for RE and RP in our study were 15–20 points lower. A previous study suggested that a difference of 3–5 points was clinically meaningful [36]; therefore, this discrepancy indicates that the family caregivers in the current study were a vulnerable group. With respect to caregiver characteristics, 57 % were women, and 76.2 % were spouses and children. These findings are consistent with results from other national studies involving caregivers for elderly people [37,38]. Also this reinforces the social and cultural roles attributed to women in terms of domestic activities and care for family members. If elderly people were unable to care for their spouses because of their own health problems, their children assumed responsibility. Providing help with daily activities and financial support was part of a natural and expected process, in reciprocation for the care they received as children, and as an act of love and respect for their parents. Further, 93.6 % of caregivers were married, similar to the findings of other related studies [5,39]. The cost of accommodation and long-term care for the elderly could exert a negative impact on caregivers’ health and the quality of care provided. Caregivers need help from the community, the government, and volunteers, to obtain spiritual and physical support, particularly with respect to medical treatment [34]. Emotional and social deprivation, which could result from ingrained cultural values and norms, is more difficult to identify and address [40,41].

### Significant Factors Associated with Family Caregivers’ QoL

#### The impact of elderly individuals’ characteristics

The age of elderly individuals was the main influential factor in the PCS. This is consistent with findings from previous studies [42,43]. Age was considered the most crucial factor studied (See Table 3). The age of elderly individuals was noted as an important factor in assessing caregivers’ health status.

Elderly individuals’ marital status and ADL scores were also influential for family caregivers in both the PCS and MCS. The elderly people’s higher dependence was associated with greater burden for caregivers [5]. As care recipients demanded more help, the average burden of daily caregiving increased. This exerted a greater impact on caregivers’ lives and work, and increased their pressure, leading to the deterioration of their own health. Older, married elderly people received greater social support and shared the burden and pressure with caregivers [39,44], which reduced the impact exerted on the caregivers’ physical and mental health.

Elderly individuals’ comorbidities were not influential factor for family caregivers in both the PCS and MCS, which was inconsistent with the results of Dauphinot who stated that the caregiver’s burden was higher when patients' comorbidities increase [45]. This related to the fact that most elderly individuals were partially dependent (72.2 %) and were suffering with more than two kinds of comorbidities (73.7 %), which need to expand the sample and analyze the data via the classification of comorbidity.

#### The impact of caregivers’ characteristics

The results showed that caregivers’ older age and additional comorbidities were risk factors with respect to the PCS and MCS; this was consistent with results of previous studies [42,43] and may be related to normal human growth and development. Young people tend to be more physically vigorous and better functioning physiologically, with less complaint for bodily pain and more energy for social activities, which provides temporary relief from the family caregiver role [43]. In addition, this energy enables people to function socially, reducing the occurrence of psychological problems, such as anxiety and depression, and contributing to the good maintenance of mental health. With increasing age, strength wanes and function of the human body deteriorate, increasing the risk of illness. Family caregivers with chronic diseases were required to endure the pain caused by their own diseases in addition to taking care of their elderly family members. This led to deterioration in their health and evoked anxiety, depression, and other negative emotions [46,47]. This phenomenon occurs more frequently in senior family caregivers. Age exerted the most direct impact on caregivers’ health; therefore, adequate attention should be paid to senior caregivers, who should be provided with appropriate assistance.

#### The impact of accommodation

Although 93.1 % of elderly in this study had a pension and pension insurance, the pension was for the payment of medical expenses for inpatient care and not for the long term care. So, 82.1 % of caregivers were required to use their own incomes to cover part of the costs of caring for elderly family members. Use of one’s own financial resources to care for an elderly person interferes with the family economy and dynamics, which could create stress and financial burden. Previous studies have shown that family caregivers’ psychological burden was significantly affected by economic pressure and led to anxiety, depression, and other psychological problems in the long term [20]. China has a growing elderly population, which requires comprehensive official arrangements for the provision of basic guarantees and services. According to the results of the study, the cost of accommodation for the elderly could affect family caregivers psychologically. The lack of assistance and economic support available for the elderly has revealed that social security and assistance services do not function successfully. In addition, governments at all levels should provide economic and administrative maintenance for this care sector, particularly for long-term care.

#### The perceived effects of caregiving on caregivers’ social lives

The perceived effects of caregiving on caregivers’ social lives constituted an important factor with respect to the MCS. The impact that care activities exert on family caregivers affects their health. Prolonged care activities impact quality of life including distress over managing complex care, disrupted social activities, depression and fatigue, withdrawal from family or friends and even lost of employment [48]. On account of a lack of pension institutions and supports, community care is currently in its infancy in China. In addition, with the impact of traditional Chinese culture, family caregivers assume major responsibility for taking care of family members. This increases economic pressure and ultimately affects family caregivers’ health. In view of these issues, caregivers should be offered services providing physical, psychological, social, and economic supports. For example, a health records information system should be established for family caregivers. Community health professionals could provide health education sessions and organize special events for family caregivers. This would increase interaction between family caregivers and allow them to establish social networks.

This study has two important implications for healthcare providers. First, positive psychological interventions and social support systems should be established to improve mental health of family caregivers. Second, governments should assume responsibility for implementing existing public policies and for extending those that target the prevention of health-related complications to promote caregivers’ health, such as financial support and establishment of facilities, especially for those who are older, have higher rates of comorbidities, are economically burdened by the elderly person’s healthcare expenses, and take care of unmarried, severely dependent elderly family members.

### Limitations

The study was subject to some limitations. A cross-sectional design was used; the current findings could be strengthened and a more accurate picture could be provided in future studies with a longitudinal design. In addition, the research involved only six communities within a single province of China. Although there were no differences between those who consented and those who declined in age and gender, the generalizability of the results is limited.

## Conclusion

Family caregivers for elderly people with chronic diseases exhibited superior PF and poorer values for the MCS, particularly with respect to RE, RP, and GH, compared with Chinese population norms. Caregiving factors and the demographic characteristics of both caregivers and the elderly contributed to caregivers’ QoL. Younger age and fewer comorbidities in caregivers as well as younger age, being married, and independent ADL status in the elderly were significantly associated with a higher PCS. Also, little importance to the perceived effects of caregiving on caregivers’ social lives was significantly associated with a higher PCS. Older age and a higher number of comorbidities in caregivers in addition to severely dependent ADL status and being unmarried in the elderly, were significantly associated with a lower MCS. Greater importance to the perceived effects of caregiving on caregivers’ social lives and capability to afford the majority of the elderly person’s healthcare expenses, were also significantly associated with a lower MCS.

These findings highlight the importance of addressing mental health and of providing financial support and social well-being for family caregivers. Furthermore, greater attention should be given to the health of caregivers who are advanced in age, burdened by higher rates of comorbidities, shouldering the elderly person’s healthcare expenses, or caring unmarried or severely dependent elderly family members. Governments at all levels should assume responsibility for implementing existing public policies, particularly with respect to the provision of financial support and establishment of facilities.

## Abbreviations

ADL, activities of daily living; BP, bodily pain; GH, general health; IADL, instrumental activities of daily living; MCS, mental component scores; MH, mental health; PCS, physical component scores; PF, physical function; QoL, quality of life; RE, role limitations due to emotional problems; RP, role limitations due to physical problems; SF, social function; SF-36, 36-item short form health survey; VT, vitality

## Additional file

Additional file 1: Table S1 Comorbidities of Caregivers and Elderly People with Chronic Diseases. (DOC 30 kb)
