# Supplementary material for: Quality of life in Chinese family caregivers for elderly people with chronic diseases
Source: Health Qual Life Outcomes. 2016 Jul 6;14:99. doi: 10.1186/s12955-016-0504-9 (PMC4936114; doi:10.1186/s12955-016-0504-9)
Supplement: Additional file 1: Table S1 — Comorbidities of Caregivers and Elderly People with Chronic Diseases. (DOC 30 kb) [file 12955_2016_504_MOESM1_ESM.doc]

Table. Comorbidities of Caregivers and Elderly People with Chronic Diseases

| Comorbidity | Caregiver | Elderly |
| --- | --- | --- |
|  | *n* (%) | *n* (%) |
| Hypertension | 46 (23.4) | 302 (29.6) |
| Coronary disease | 9 (4.6) | 101 (9.9) |
| Diabetes mellitus | 32 (16.2) | 98 (9.6) |
| Rheumatic arthritis | 21 (10.7) | 97 (9.5) |
| Cerebrovascular disease | 4 (2.0) | 94 (9.2） |
| Osteoporosis | 18 (9.1) | 89 (8.7) |
| Hyperlipidemia | 33 (16.7) | 84 (8.2) |
| Others (Alzheimer's disease, asthma, chronic obstructive pulmonary disease, peptic ulcer, etc) | 34 (17.3) | 154 (15.3) |
